# The rise of testicular germ cell tumours: the search for causes, risk factors and novel therapeutic targets

**DOI:** 10.12688/f1000research.2-55.v1

**Published:** 2013-02-19

**Authors:** Skye C McIver, Shaun D Roman, Brett Nixon, Kate L Loveland, Eileen A McLaughlin

**Affiliations:** 1 ARC Centre of Excellence in Biotechnology & Development, School of Environmental & Life Sciences, University of Newcastle, Callaghan, 2308, Australia; 2 Department of Biochemistry & Molecular Biology, School of Biomedical Sciences, Monash University, Clayton, 3800, Australia; 3 Department of Anatomy & Developmental Biology, School of Biomedical Sciences, Monash University, Clayton, 3800, Australia

## Abstract

Since the beginning of the 20th century there has been a decline in the reproductive vitality of men within the Western world. The declining sperm quantity and quality has been associated with increased overt disorders of sexual development including hypospadias, undescended testes and type II testicular germ cell tumours (TGCTs). The increase in TGCTs cannot be accounted for by genetic changes in the population. Therefore exposure to environmental toxicants appears to be a major contributor to the aetiology of TGCTs and men with a genetic predisposition are particularly vulnerable. In particular, Type II TGCTs have been identified to arise from a precursor lesion Carcinoma *in situ *(CIS), identified as a dysfunctional gonocyte; however, the exact triggers for CIS development are currently unknown. Therefore the transition from gonocytes into spermatogonia is key to those studying TGCTs. Recently we have identified seven miRNA molecules (including members of the miR-290 family and miR-136, 463* and 743a) to be significantly changed over this transition period. These miRNA molecules are predicted to have targets within the CXCR4, PTEN, DHH, RAC and PDGF pathways, all of which have important roles in germ cell migration, proliferation and homing to the spermatogonial stem cell niche. Given the plethora of potential targets affected by each miRNA molecule, subtle changes in miRNA expression could have significant consequences e.g. tumourigenesis. The role of non-traditional oncogenes and tumour suppressors such as miRNA in TGCT is highlighted by the fact that the majority of these tumours express wild type p53, a pivotal tumour suppressor usually inactivated in cancer. While treatment of TGCTs is highly successful, the impact of these treatments on fertility means that identification of exact triggers, earlier diagnosis and alternate treatments are essential. This review examines the genetic factors and possible triggers of type II TGCT to highlight target areas for potential new treatments.

## Introduction

Testicular cancers are generally grouped into three broad categories with type I testicular germ cell tumours (TGCTs) being observed primarily in neonatal boys and young children and consisting of benign teratomas and malignant yolk sac tumours ^1^. Type III TGCT, also called spermatocytic seminomas, affect older men above 50 years of age and are derived from a slow growing expansion of type B spermatogonia ^2^. Type II TGCTs mostly affect men aged between 20 and 40; however, the origins of this tumour type are much earlier during foetal development ^3^. All type II TGCTs develop from a pre-invasive lesion termed Carcinoma *in situ* (CIS), which has been identified as a dysfunctional foetal germ cell ^2, 4^.

In the developed world the incidence of type II TGCT (seminoma and non-seminoma), but not type I or II, has increased significantly over the last century to become the most common malignancy found in men aged between 20 and 40 years of age ^5– 7^. Such findings have led to speculation that environmental factors impact on the tumorigenesis of this cancer ^8^. In addition, several genetic mutations have been discovered that positively impact TGCT rates in both sporadic and familial cases of TGCTs ^8, 9^. While the characterisation of risk factors is far from being completed, one of the most promising indicators of TGCT is mutations in *c-KIT* and *KIT ligand* ^10– 13^.

The perceived association of TGCTs with infertility combined with evidence that pre-existing subfertility increases in patients with type II tumours, raises the possibility that these lesions may act as indicators of a general reduction in male reproductive health and rising fertility problems within the male population ^14, 15^. Type II TGCT is known to develop from dysfunctional gonocytes located within the seminiferous tubules ^16^, indicating that the risk factors predisposing an individual to TGCTs are active early in male foetal development, necessitating the investigation of early germ cell development in an effort to identify causative factors for type II TGCTs. In this review we examine normal germ cell development and potential areas of this differentiation that are modified during the development of type II TGCTs. We also discuss the genetic and environmental contributions to disease specification as well as risk factors and indicators of tumour progression.

## Germ cell development

### Germ cell specification

**Figure 1.**
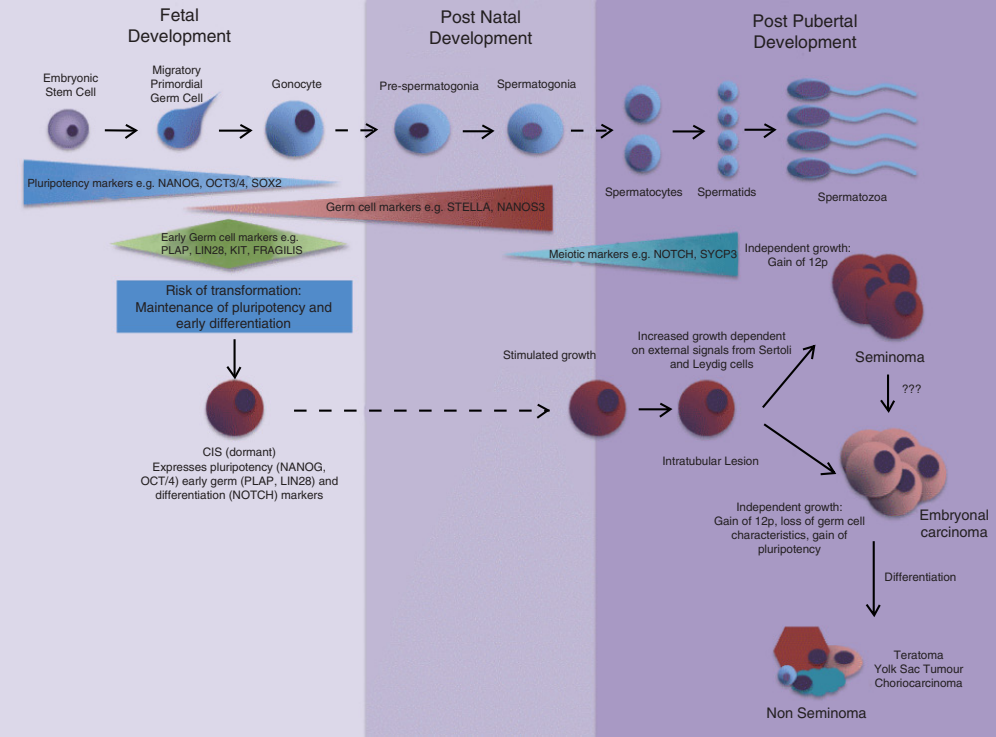
Proposed mechanism of type II testicular germ cell tumour (TGCT) differentiation. This is an update and adaption of the classic model of type II TGCT development first proposed by Rajpert-De Meyts ^93^. Current understanding indicates maintained pluripotency combined with incomplete premature differentiation of gonocytes causes the specification of Carcinoma *in situ* (CIS) cells. Signals caused by puberty cause these cells to proliferate and once additional mutations accumulate CIS cells differentiate into overt type II TGCTs.

Primordial germ cells (PGCs) are signalled to develop by Bone Morphogenic proteins (BMPs) secreted from the extraembryonic ectoderm and the visceral endoderm ^17^. BMPs (BMP4, BMP2, and BMP8b), in turn activate the expression of Fragilis (IFITMS) genes ^17, 18^. In the mouse around six of the Fragilis positive cells begin to express BLIMP1/PRDM1 and PRDM14 at embryonic day (E) 6.5 and thereafter are committed to the germ line ( Figure 1) ^19– 23^. BLIMP1 is a key component of germ cell specification as studies of the BLIMP1 knockout mouse have revealed that PGCs cluster, but fail to migrate ^24^. The expression of BLIMP represses the expression of somatic genes such as HOX, FDF8 and SNAIL, in addition to DNA methyl transferases ^25^. The combination of the highly proliferative nature of the germ cells i.e. expansion from six cells at E6.5, to 250 cells at E9.5, 1000 cells at E10.5, and 26,000 cells at E13.5, and the lack of maintenance DNA methyltransferases causes general demethylation of the DNA in primordial germ cells ^25, 26^. The methylation status, and the repression of somatic cell phenotype, permits the expression of pluripotency factors such as NANOG and OCT 3/4 (but not c-MYC and KLF - required for total pluripotency) ^26^. SOX2, an essential pluripotency factor in mouse, is not expressed in human primordial germ cells. However, it is expressed in nonseminoma germ cell tumours ^26^.

OCT 3/4 is expressed in pluripotent cells of the inner cell mass of a blastocyst and, in contrast to the differentiating somatic cells, its expression is maintained in primordial germ cells ^17, 27^. OCT 3/4 is believed to control primordial germ cell survival, given that germ cells are lost through an apoptotic pathway in OCT 3/4 null mice ^17, 28^. In mice, SOX2 acts in conjunction with OCT 3/4 and its expression is maintained in primordial germ cells until they migrate, colonise the gonad, and become specified as pre-spermatogonia or oogonia, whereupon SOX2 is down regulated ^17, 29^. The pattern of NANOG expression tracks that of SOX2, and is required for the maturation of germ cells once they reach the genital ridges ^17, 30^. The exact roles of OCT 3/4, SOX2 and NANOG in primordial germ cells is currently unknown, but as they are important for maintaining pluripotency and proliferation in embryonic stem (ES) cells, it is considered likely that they also maintain pluripotency in germ cells and prevent their differentiation ^17^. More recently, several other pluripotency factors have been identified in primordial germ cells, gonocytes and pre-spermatogonia ^20^. One such protein is LIN28, which is involved in maintaining pluripotency and survival. LIN28 is located upstream of, but linked to, both OCT 3/4 and NANOG expression. Additionally LIN28 is not found in postnatal spermatogonia but is expressed in CIS, seminoma, and embryonal carcinoma, further demonstrating the importance of the differentiation of germ cells to maintain normal testis development ^20, 31^. Another pluripotency protein identified upstream of the classical pluripotency regulator OCT 3/4, and capable of interacting with NANOG, is SALL4 ^32^. SALL4 is expressed late primordial germ cells, pre-spermatogonia, and spermatogonial stem cells and is hypothesised to be involved in both the maintenance of primordial germ cells as well as the differentiation of spermatogonial stem cells ^32, 33^.

### Primordial germ cells and pluripotency

Interestingly, primordial germ cells are not natively pluripotent. Indeed, despite the fact that they express many pluripotency markers, they only differentiate into one cell type i.e. a germ cell ^26^. However, pluripotency can be induced in *in vitro* cultures of germ cells, if they are isolated before the colonisation of the gonad and incubated in the presence of the growth factors SCF, FGF2, and LIF ^17^. In addition, germ cells are more efficiently transformed into pluripotent cells in the presence of FFG2 in conjunction with MAK2k and GSK3B, as well as TGFB type 1 receptor inhibitors ^34^.

### Germ cell maintenance

Once primordial germ cells are specified, germ cell specific genes that promote cell survival, such as STELLA and NANOS3, are up-regulated ( Figure 1) ^17^. Other markers of primordial germ cells include SSEA1, PRDM14, DND1, Fragilis, LIN28, c-KIT and MVH ^26^. DND (dead end/Ter) prevents miRNA mediated translational repression and serves as a survival factor for PGCs. Mutations in DND cause testicular teratomas and DND null mice lose their PGCs via apoptosis between E8.5 and E12.5 ^9^. At E7.5 in the mouse (3 weeks in humans) PLAP (Placental Like Alkaline Phosphatase)-positive PGCs reside in the posterior of the primitive streak and become motile shortly after this time ^35, 36^.

### Germ cell migration

Primordial germ cells initially migrate into the hindgut during its anterior extension (E8-9.5); they then move into the mesoderm (E9.5) and bilaterally travel to the genital ridges to contribute to the formation of the gonads (E10.5-11.5) ^19, 37^. This process is complete by E33-37 in humans ^37, 38^.

Steel factor (KIT-ligand) has been identified as a key survival and proliferative signal for developing germ cells as well as acting to guide PGCs along the hindgut and towards the genital ridges ^39, 40^. The movement of PGCs out of the hindgut and into the gonads (E9.5) is dependent on E-cadherin (CDH1) and β1-integrin (ITGB1) ^37^, and is directed by CXCL12 ^41^. On reaching the genital ridges at around E11 to E11.5, the PGCs proliferate and form gonocytes ^35^. At this time, active demethylation continues by UTX (histone demethylase), another pluripotency factor, before the cells then undergo sex specific epigenetic changes required to produce viable germ cells ^25, 42^. By E13.5 the gonocytes enter either mitotic arrest in the case of testis, or meiotic arrest in the ovary. Therefore the period of time between the arrival in the gonad and arrest is key to the primordial germ cell proliferation and differentiation ^43^.

### Sex determination

Male sex determination is triggered by the expression of SRY (Sex-determining region on the Y chromosome), a high mobility group (HMG) transcription factor which activates SOX9 (SRY related HMG box 9), another transcription factor which in itself is sufficient for sex determination ^44, 45^. SOX9-positive pre-Sertoli cells recruit cells from the mesonephros and the coelomic epithelium to form the testicular cords ^46, 47^ which occurs in concert with the commitment of male germ cells to the pre-spermatogonia cell fate ^48^. Sertoli cells also secrete paracrine factors (DHH and platelet-derived growth factors) initiating the differentiation of the testosterone producing Leydig cells ^49^.

Male germ cells are maintained in mitotic arrest within the seminiferous tubules by the enzyme CYP26B1 which facilitates degradation of retinoic acid, preventing the expression of STRA8 (stimulated by retinoic acid 8) and hence entry into meiosis ^50^. When the expression of CYP26B1 decreases at E13.5, the RNA binding protein NANOS2 maintains mitotic arrest in male germ cells ^51^. Shortly after birth in mice, and in late gestation in humans, gonocytes (prospermatogonia) migrate from the centre to the basement membrane of the seminiferous tubules and by postnatal day 6 they have begun to divide and are designated single spermatogonia (A _s_) or spermatogonial stem cells (SSCs) and spermatogenesis is initiated ^52^.

## Risk factors and genetic predisposition of testicular cancer

The risk factors for type II TGCTs include family predisposition, cryptorchidism, disorders of sexual development, high maternal oestrogen during foetal development, environmental exposures, sub- or infertility, and previous TGCTs ^3, 36, 53^. Additionally, geographical regions with a high rate of TGCTs have lower sperm quality as well as increased rates of cryptorchidism and hypospadias when compared to regions with low rates of TGCTs ^54^. While not all sexual development disorders lead to an increased risk of TGCTs, they usually occur at higher frequency in less differentiated gonads ^55^. Identified genetic factors, including deletions on the Y chromosome, androgen insensitivity, and KIT mutations; negatively impact germ cell development supporting the notion that the risk of TGCTs is established early ^3, 36, 56^. It is proposed that the combination of both genetic and environmental factors prevents or delays the maturation of PGCs/gonocytes into pre-spermatogonia by retarding the development of the supporting Sertoli and Leydig cells, thus perturbing the microenvironment required for germ cell development ^3, 57^.

Patients lacking the SRY gene on the Y chromosome required for Sertoli cell development, or WT1 required for ovary development, have a high risk of type II TGCTs, because of the lack of correctly differentiated support cells, which in turn inhibit germ cell development ^3^. A partial deletion of the AZoospermia Factor C (AZFC) region (gr/gr mutation) on the short arm of the Y chromosome is also associated with an increased risk of TGCTs ^56^.

Mutations in KIT signalling and continued KIT expression are seen in CIS cells, but not more advanced stages of testicular cancer, indicating that this may be an initial feature of CIS cell specification. Furthermore, genetic association studies have identified mutations in genes encoding KIT, KIT ligand and its downstream signalling molecules such as KRAS, SPRY4, and BAK1, as likely predisposition genes for TGCTs ^3, 58– 60^. It is believed that a region of the Y chromosome encoding TSPY (testis specific protein Y-encoded), with a potential role in germ cell mitotic division, is required for the development of TGCTs. Interestingly, as with KIT, TPSY protein expression is lost when CIS becomes invasive ^36, 55^, suggesting that it may have a role in the initiation, but not maintenance, of tumours.

The switch to meiosis from mitotic amplification is tightly controlled in normal spermatogenesis and there is some indication that this pathway may be prematurely initiated then aborted in CIS and TGCTs ^61^. Several members of the NOTCH signalling pathway control the mitotic to meiotic switch, and mutations in the NOTCH signalling pathway in *C. elegans* have been shown to elicit tumour-like expansion of germ cells ^61^. This observation stimulated exploration of NOTCH signalling in human TGCTs and revealed that NOTCH1 and NOTCH4 are both over-expressed in CIS and seminoma. Additional genetic screening of affected individuals has identified mutations in members of the NOTCH family as primary risk factors for developing these pathologies ^61, 62^. The meiotic protein SYCP3, a component of the synaptonemal complex which aligns sister chromatids in prophase I of meiosis allowing their proper segregation, is also expressed in CIS, seminoma and embryonal carcinoma, thus providing further indication of a premature activation of meiosis in TGCTs development ^61^.

More recent genetic screens have identified transcription factors DMRT1 and ATF7IP, as well as the telomere regulator TERT, as susceptibility genes for TGCTs ^58^. DMTR1 is required for normal testicular differentiation and DMTR1 knockout mice have been shown to develop teratomas ^58^. ATF7IP is a transcription factor that regulates the expression of subunits of the enzyme complex responsible for maintaining telomere length such as the active component, TERT ^63^. Telomere regulation and length is tightly controlled within cells and usually only immortal cells, i.e. stem cells, express active telomerase. Telomerase is also extensively overexpressed in cancer cells and its activity plays a key role in the transformation process ^63, 64^.

However, in most cases no known genetic factor contributing to the development of TGCTs is identified and therefore environmental factors may play an essential role in the development of tumours in these individuals. The rates of TGCTs within Western countries have risen sharply since the early 1900s; however, this rise is not consistent across all countries ^8, 65^. The most notable differences in TGCT incidences and rates of increase are demonstrated in Europe. For example, increased TGCT rates were documented in England, Wales, and Germany before Denmark and Norway. By contrast, Finland, Eastern Germany, and Poland, still have lower TGCT rates than surrounding countries ^65, 66^. The rapid rise in TGCT rates, as well as the variation between geographical locations, implicates exposure to one or more environmental toxicants as a key risk factor ^65^. For instance, it is believed that xeno-oestrogens and anti-testosterones negatively affect the development of Sertoli cells and Leydig cells causing a suboptimal environment for germ cell differentiation leading to reduced fertility and development of CIS cells ^3^. Phthalates represent another possible environmental toxicant that could contribute to the incidence of TGCTs. Indeed, exposure to these toxins is known to be highly variable between different countries and this could, in part, contribute to geographical differences in cancer rates ^65^. Additionally, high levels of phthalates have been documented in the blood of mothers whose sons developed TGCT ^6^. Similarly, rats exposed to phthalates exhibit symptoms similar to testicular dysgenesis, which is also associated with a high risk of TGCT ^65^. The possibility of toxicant exposure leading to TGCT has led to the investigation of detoxification mechanisms in cancer sufferers and their mothers ^56^. While the power of this study is compromised by the small sample size, the analysis did demonstrate a link between specific cytochrome p450 polymorphisms and an increased rate of TGCT development ^56^.

## The development of type II testicular germ cell tumours

TGCTs originate from CIS cells, which are found on the basement membrane of abnormal seminiferous tubules that usually lack normal spermatogenesis. CIS cells were first identified in 1972 in biopsies of infertile men ^67^, and a causal link was subsequently established when, during follow-up, the men with this lesion developed tumours while control subjects exhibited no cases of TGCT ^53^. Furthermore, the incidence of CIS is about equal to that of type II TGCTs, suggesting that CIS cells will eventually develop into overt TGCT ^36^.

Morphologically, CIS cells resemble primordial germ cells/gonocytes and express a number of similar markers, in that they lack imprinting and express OCT 3/4, PLAP and c-KIT ^36, 68^. In fact, the transcriptome of CIS cells is extremely similar to that of isolated normal human gonocytes, demonstrating a close relationship between these cell types ^16^. CIS cells appear to be blocked from differentiating and entering spermatogenesis ^3^. Instead they accumulate within the seminiferous tubule to become an intratubular lesion termed either seminoma or non-seminoma (embryonal carcinoma) ^3, 36^. Interestingly, seminomas can later reprogram to become non-seminomas ^36^. These intratubular lesions proliferate to fill the lumen of the seminiferous tubule and rely on Sertoli cell-derived growth/survival signals and Leydig cell production of testosterone ^36^. Once these cells break their dependence on external signals they become overt testicular cancer ^36^.

Invasive TGCTs are characterised by a gain in copy number of the short arm of chromosome 12 ^3, 53^. Within this chromosomal region there are several genes encoding candidate virulence factors, including NANOG, KRAS2 and BCAT1; however, their role has yet to be confirmed ^3^. BCAT1 for example, is only expressed in embryonal carcinoma and not in other germ cell lesions, indicating that the role of the genes on chromosome 12 may vary depending on the tumour type ^3^. There are several markers which identify seminoma and non-seminoma tumours; for example, OCT 3/4 and NANOG are expressed in both seminoma and embryonal carcinoma, while SOX17 is specific to seminoma tumours and SOX2 is only expressed in non-seminoma ^69^. In extended culture, ES cells gain excess copies of chromosome 12, but they do not seem to exhibit malignant characteristics, indicating that the gain of chromosome 12 is not the sole initiation factor for virulence in TGCTs ^3^.

## Possible mechanisms of germ cell cancer specification

Several risk factors have been identified in a mouse model of teratoma formation (strain 129/Ter) ^70^. These include the continued expression of pluripotency markers and proliferation as well as premature differentiation, e.g. precocious entry into meiosis. Usually pluripotency factors such as NANOG, SOX2, and OCT 3/4, are down-regulated following cell cycle arrest at E13.5, but the germ cells of Ter mice continue to proliferate and express NANOG at E15.5 ^70^. In this mouse model, PGCs also appear to prematurely differentiate with the detection of cyclin D1 as early as E13.5 ^70^, compared to its normal expression in spermatogonia at postnatal day 4 ^71^. It is possible that a similar mechanism occurs for the development of CIS cells considering that CIS cells express markers for primordial germ cells including PLAP, OCT 3/4, and c-KIT, long past embryonic development ^3^. Concurrent with this delayed maturation is the expression of meiotic genes including NOTCH and SYCP3 in both CIS cells as well as seminoma cells ^61^. Therefore maintenance of pluripotency in primordial germ cells while undergoing a defective maturation process, which may include premature activation of meiosis, could underpin the specification of these cells as CIS and tumorigenic ( Figure 1).

## Metastasis

Patients with stage I TGCTs and a concomitant high risk of metastasis are destined to undergo aggressive surgery and chemotherapy ^3^. So far, vascular invasiveness, percentage of embryonal carcinoma and the proliferation index, have been the best predictors of metastasis risk. However, more recently the chemokine-mediated CXCR4 pathway has demonstrated some promise in metastasis prediction - with tumours containing localised high CXCL12 expression being less likely to metastasise ^3, 72^. Gilbert and colleagues ^72^ examined TGCTs and found that seminomas expressed higher levels of CXCR4 transcript and protein, than normal testis, but this trend was not maintained in non-seminomas. In addition Gilbert *et al.* ^72^ demonstrated that a seminoma cell line (TCam2) migrates in response to CXCL12α, via activation of the MAP kinase pathway. However, the non-seminoma cell line 2102EP does not, which is not surprising, as this cell line does not express CXCR4. We have independently looked at the expression of CXCL12 and CXCR4 in TGCTs (McIver SC, Loveland KL, Roman SD, Nixon B, Kitazawa R, McLaughlin EA, unpublished observations) and confirmed that CXCR4 mRNA was overexpressed in seminomas. However, at the protein level we did not find elevated levels of CXCR4 when its expression was examined via immunohistochemistry. Additionally, the tight correlation between CXCL12/CXCR4 with MAP kinase activation that is found in normal testis was abolished in the TGCT samples. We were able to confirm that both CXCL12α and β caused cell migration in the seminoma cell line (TCam2) while no migration response was observed in the CXCR4 positive non-seminoma cell lines 833ke and NTera2/D1. Therefore the expression of CXCL12 and CXCR4 is more likely to be a better indicator of the possibility of seminoma metastasis rather than non-seminoma.

## Treatment resistance

Generally seminoma cells (a category that includes CIS cells) are extremely sensitive to irradiation as well as cisplatin-based chemotherapy drugs, while non-seminomas only respond to chemotherapy ^3^. However, the mature teratoma components of non-seminoma tumours are resistant to DNA damage therapies, which is consistent with the loss of embryonal cell characteristics these tumour exhibit ^3^. Although the common chemotherapy and radiotherapy treatments are very effective and maintain quality of life, they can lead to infertility, hypogonadism and retrograde ejaculation, which new treatment options should seek to avoid ^2, 15^. Both seminoma and non-seminoma tumours can become resistant to traditional oncology treatments ^3^. Interestingly, evidence suggests that the methylation status of the cells DNA controls the expression of specific genes, such as c-FLAR, which inhibits caspase-dependant apoptosis, thus imparting resistance to cisplatin treatment ^3^. Several other genes have been implicated in cisplatin resistance including Cyclin D1 ^3^. Cyclin D1 is expressed in murine germ cells from post natal day 3, which coincides with the appearance of spermatogonia in testis and therefore may control the proliferation and differentiation of these cells ^71^. The differentiation and abnormal proliferation of gonocytes is essential to the development of CIS, and therefore Cyclin D1 may play a key role in CIS evolution.

## Role of miRNA in testicular germ cell tumours

A particularly exciting recent finding is that different types of TGCTs can be distinguished on the basis of their distinct miRNA expression profiles. For example, miR-122a is only expressed in yolk sac tumours ^69, 73^. Also the regulator of miRNA maturation, LIN28, has been shown to be expressed in PGCS, gonocytes and pre-spermatogonia CIS, seminoma and non-seminoma, where it regulates totipotency, and functions upstream of the tumour/pluripotency transcription factors OCT1/3 and NANOG ^31^.

The tumour suppressor p53 generally controls the exit from the cell cycle upon DNA damage, to allow for either repair or apoptosis. It follows that p53 mutation is usually a key step in tumorigenesis; however, in TGCTs p53 is only rarely inactivated ^74^. Voorhoeve *et al.* ^75^ provided an explanation for this phenomenon when they determined that the presence of microRNAs; miR-372 and miR-373; conveyed a growth advantage and protected against the cellular senescence response to DNA damage, in the presence of wild type p53. Analysis of the molecular basis for this effect revealed that these miRNA molecules act in conjunction with RAS downstream of p53 in order to mimic the phenotype of inactivated p53 ^75^. Furthermore, the authors identified LATS2 (Large tumour suppressor homology 2) as a potential target of p53 signalling given that LATS2 deletion accelerates cellular proliferation and tumour development. This can be phenocopied in some seminomas, which do not overexpress miR-372 or miR-373, by mutations in LATS2. The LATS2 protein inhibits cyclin E/CDK2 activity and thereby arrests the cell cycle ^75^. Later studies found that this miRNA cluster is exclusively over expressed in almost all seminomas and most embryonal carcinoma tumours ^74^.

In addition to their role in the tumorigenesis of TGCTs, miRNAs have an essential role in the development of primordial germ cells and in spermatogenesis ^76^. Therefore we decided to examine the change in the miRNA expression profile between postnatal gonocytes and spermatogonia in mice in the hope of gaining a better understanding of this essential step in both normal spermatogenesis and CIS development. This approach led to the identification of seven differentially expressed miRNA molecules (three of which were up regulated; miR-136, -743a and -463*, and four down regulated miR290-5p, -291a-5p, -294* and -293) during this developmental phase. Analysis of their potential targets indicated that these miRNA molecules were likely to impact the PTEN and Wnt/β catenin signalling pathways ^77^ as well as the CXCR4 signalling pathway previously implicated in TGCT metastasis (McIver SC, Loveland KL, Roman SD, Nixon B, Kitazawa R, McLaughlin EA, unpublished observations). The PTEN tumour suppressor is known to inhibit PI3K signalling to negatively regulate cell growth and therefore its suppression causes increased proliferation and potentially tumourigenesis. The loss of PTEN is also associated with the transformation of CIS cells into overt cancerous tumours ^78^. Furthermore, increased relapse rate in TGCTs has also been found to be associated with the loss of PIK3IP1, an additional negative regulator of PIK kinase ^79^. Both the PTEN and Wnt/β catenin pathways converge to control the function of Cyclin D1, which is expressed in highly proliferative TGCTs where down-regulation instigates cell cycle arrest ^80^, and is associated with chemotherapy resistance in these tumours ^3^. Indeed, on this basis, the control of Cyclin D1 activity has been suggested as a potential treatment target ^80^. Subsequently we selected the three most highly differentially expressed miRNA (Mir-743a, -291a-5p, -293) and instigated a strategy to directly identify their targets. The use of a pull down approach pioneered by Orum and Lund ^81^ in which the miRNAs were used as bait, and bound mRNA analysed by microarray, revealed a significant number of predicted and non-predicted targets bound to each miRNA (McIver SC, Roman SD, Nixon B, McLaughlin EA., unpublished observations).

Interestingly the targets identified were postulated to regulate key germ cell migration and proliferation pathways controlled through PDGF ^82^ and RAC ^83^ signalling (McIver SC, Roman SD, Nixon B, McLaughlin EA, unpublished observations). PDGF signalling is known to play a role in gonocyte migration and proliferation ^82, 84^. In addition, PDGF signalling has been shown to work in conjunction with oestrogen signalling ^84^, a mechanism that is easily hijacked by the presence of xeno-oestrogens. In fact gonocytes have been shown to be more sensitive to xeno-oestrogens than endogenous ligands ^84^. This has implications for the development of CIS because high concentrations of xeno-oestrogens are associated with increased TGCT risk ^3^. RAC on the other hand has been shown to be essential for the transmigration of spermatogonial stem cells through the blood-testis barrier during testis colonisation assays ^83^. Using a knockdown assay as an alternate technique to determine the targets of miR-291a-5p, -293 and -743a we identified one promising gene, IGFBP7 (Insulin-like growth factor binding protein 7) (McIver SC, Roman SD, Nixon B, McLaughlin E A, unpublished observations), which is known to act as either a positive (e.g. in glioma ^85^) or negative regulator (e.g. in breast and liver cancer ^86, 87^) of cell proliferation and migration depending on the environment in which it is found. Therefore the disrupted expression of IGFBP7 by aberrantly expressed miRNAs during the gonocyte to spermatogonia transition could have implications for their growth and differentiation.

The miRNA molecules found to be down regulated during gonocyte differentiation into spermatogonia all belong to the miR-290 family. This cluster of miRNA is highly expressed in embryonic stem cells (ES) and is known to be a key regulator of ES cell pluripotency, controlling the expression of OCT4, SOX2 and NANOG ^88– 90^. Additionally, upon knockout of the miR-290 cluster, aberrant migration of germ cells has been observed with significantly fewer primordial germ cells reaching the bipotential gonad ^88^. Comparatively less is known about the miRNA molecules that were found to be upregulated during gonocytes differentiation. miR-136 is proposed to be a tumour suppressor in glioma and is capable of targeting the anti-apoptosis genes AEG-1 and BCL-2 ^91^. miR-743a on the other hand is involved in oxidative stress responses ^92^. On the basis of these data, as well as our own observations of miRNA involvement in key germ cell regulatory pathways ^77^, it appears likely that the changing miRNA profile between postnatal gonocytes and spermatogonia could have a fundamental role in the development of CIS within the testis.

## Concluding remarks

Research into both the genetic and environmental factors that predispose an individual to type II TGCTs has been hampered by the lack of a suitable animal model for the study this tumour type. Mouse models are suitable for the study of type I neonatal TGCTs and canines exhibit type III TGCTs caused by an expansion of type B spermatogonia. These models offer a number of advantages in terms of allowing genetic screens and treatments to be conducted to enhance our understanding of these cell types ^36^. In contrast, the study of seminoma and non-seminoma tumours is restricted to cell lines and, in the case of seminoma, these are scarce ^36^, severely limiting explorative experiments to study these tumour types. Type II TGCTs have been extensively characterised at the molecular level resulting in the identification of several predisposition genes, tumour markers, as well as the mode of tumour progression ^3^. However, despite our extensive knowledge of mature tumours, the exact triggers for the development of CIS cells remain unknown. Unfortunately, until these mechanisms are identified, therapeutic interventions are limited. Current treatment techniques are very effective but their side effects often include a loss of fertility, a concerning fact given that these tumours primarily arise in men of reproductive age ^15^. Therefore, a better understanding of the cellular mechanisms underlying germ cell development, are vital to establish novel treatments that are capable of preserving fertility in patients.

## References

[1] Frazier AL Weldon C Amatruda J: Fetal and neonatal germ cell tumors. *Semin Fetal Neonatal Med.* 2012;17(4):222–230 10.1016/j.siny.2012.05.004 22647545

[2] Bahrami A Ro JY Ayala AG: An overview of testicular germ cell tumors. *Arch Pathol Lab Med.* 2007;131(8):1267–1280 17683189 10.5858/2007-131-1267-AOOTGC

[3] Looijenga LH Gillis AJ Stoop H: Dissecting the molecular pathways of (testicular) germ cell tumour pathogenesis; from initiation to treatment-resistance. *Int J Androl.* 2011;34(4 Pt 2):e234–e251 10.1111/j.1365-2605.2011.01157.x 21564133

[4] Kristensen DM Sonne SB Ottesen AM: Origin of pluripotent germ cell tumours: the role of microenvironment during embryonic development. *Mol Cell Endocrinol.* 2008;288(1–2):111–118 10.1016/j.mce.2008.02.018 18420341

[5] Baade P Carriere P Fritschi L: Trends in testicular germ cell cancer incidence in Australia. *Cancer Causes Control.* 2008;19(10):1043–1049 10.1007/s10552-008-9168-z 18478339

[6] Skakkebaek NE Rajpert-De Meyts E Jorgensen N: Testicular cancer trends as 'whistle blowers' of testicular developmental problems in populations. *Int J Androl.* 2007;30(4):198–204 10.1111/j.1365-2605.2007.00776.x 17705804

[7] Walschaerts M Huyghe E Muller A: Doubling of testicular cancer incidence rate over the last 20 years in southern France. *Cancer Causes Control.* 2008;19(2):155–161 10.1007/s10552-007-9081-x 18236173

[8] Richiardi L Pettersson A Akre O: Genetic and environmental risk factors for testicular cancer. *Int J Androl.* 2007;30(4):230–240 10.1111/j.1365-2605.2007.00760.x 17488341

[9] Matin A Nadeau JH: Search for testicular cancer gene hits dead-end. *Cell Cycle.* 2005;4(9):1136–1138 10.4161/cc.4.9.1992 16082220

[10] Ferlin A Pengo M Pizzol D: Variants in KITLG predispose to testicular germ cell cancer independently from spermatogenic function. *Endocr Relat Cancer.* 2012;19(1):101–108 10.1530/ERC-11-0340 22194441

[11] Goddard NC McIntyre A Summersgill B: KIT and RAS signalling pathways in testicular germ cell tumours: new data and a review of the literature. *Int J Androl.* 2007;30(4):337–348 10.1111/j.1365-2605.2007.00769.x 17573850

[12] Kanetsky PA Mitra N Vardhanabhuti S: Common variation in KITLG and at 5q31.3 predisposes to testicular germ cell cancer. *Nat Genet.* 2009;41(7):811–815 10.1038/ng.393 19483682 PMC2865677

[13] Kemmer K Corless CL Fletcher JA: KIT mutations are common in testicular seminomas. *Am J Pathol.* 2004;164(1):305–313 10.1016/S0002-9440(10)63120-3 14695343 PMC1602213

[14] Aschim EL Haugen TB Tretli S: Subfertility among parents of men diagnosed with testicular cancer. *Int J Androl.* 2008;31(6):588–594 10.1111/j.1365-2605.2007.00813.x 17822418

[15] Paduch DA: Testicular cancer and male infertility. *Curr Opin Urol.* 2006;16(6):419–427 10.1097/01.mou.0000250282.37366.d2 17053522

[16] Sonne SB Almstrup K Dalgaard M: Analysis of gene expression profiles of microdissected cell populations indicates that testicular carcinoma *in situ* is an arrested gonocyte. *Cancer Res.* 2009;69(12):5241–5250 10.1158/0008-5472.CAN-08-4554 19491264 PMC2869030

[17] Western P: Foetal germ cells: striking the balance between pluripotency and differentiation. *Int J Dev Biol.* 2009;53(2–3):393–409 10.1387/ijdb.082671pw 19412894

[18] Lange UC Adams DJ Lee C: Normal germ line establishment in mice carrying a deletion of the Ifitm/Fragilis gene family cluster. *Mol Cell Biol.* 2008;28(15):4688–4696 10.1128/MCB.00272-08 18505827 PMC2493357

[19] Tarbashevich K Raz E: The nuts and bolts of germ-cell migration. *Curr Opin Cell Biol.* 2010;22(6):715–721 10.1016/j.ceb.2010.09.005 20947321

[20] Aeckerle N Eildermann K Drummer C: The pluripotency factor LIN28 in monkey and human testes: a marker for spermatogonial stem cells? *Mol Hum Reprod.* 2012;18(10):477–488 10.1093/molehr/gas025 22689537 PMC3457707

[21] Hayashi K de Sousa Lopes SM Surani MA: Germ cell specification in mice. *Science.* 2007;316(5823):394–396 10.1126/science.1137545 17446386

[22] McLaren A Lawson KA: How is the mouse germ-cell lineage established? *Differentiation.* 2005;73(9–10):435–437 10.1111/j.1432-0436.2005.00049.x 16351686

[23] Saga Y: Mouse germ cell development during embryogenesis. *Curr Opin Genet Dev.* 2008;18(4):337–341 10.1016/j.gde.2008.06.003 18625315

[24] Ohinata Y Payer B O'Carroll D: Blimp1 is a critical determinant of the germ cell lineage in mice. *Nature.* 2005;436(7048):207–213 10.1038/nature03813 15937476

[25] De Felici M: Nuclear reprogramming in mouse primordial germ cells: epigenetic contribution. *Stem Cells Int.* 2011;2011:425863 10.4061/2011/425863 21969835 PMC3182379

[26] Pirouz M Klimke A Kessel M: The reciprocal relationship between primordial germ cells and pluripotent stem cells. *J Mol Med (Berl).* 2012;90(7):753–761 10.1007/s00109-012-0912-1 22584374

[27] Yabuta Y Kurimoto K Ohinata Y: Gene expression dynamics during germline specification in mice identified by quantitative single-cell gene expression profiling. *Biol Reprod.* 2006;75(5):705–716 10.1095/biolreprod.106.053686 16870942

[28] Kehler J Tolkunova E Koschorz B: Oct4 is required for primordial germ cell survival. *EMBO Rep.* 2004;5(11):1078–1083 10.1038/sj.embor.7400279 15486564 PMC1299174

[29] Maldonado-Saldivia J van den Bergen J Krouskos M: Dppa2 and Dppa4 are closely linked SAP motif genes restricted to pluripotent cells and the germ line. *Stem Cells.* 2007;25(1):19–28 10.1634/stemcells.2006-0269 16990585

[30] Chambers I Silva J Colby D: Nanog safeguards pluripotency and mediates germline development. *Nature.* 2007;450(7173):1230–1234 10.1038/nature06403 18097409

[31] Gillis AJ Stoop H Biermann K: Expression and interdependencies of pluripotency factors LIN28, OCT 3/4, NANOG and SOX2 in human testicular germ cells and tumours of the testis. *Int J Androl.* 2011;34(4 pt 2):e160–e174 10.1111/j.1365-2605.2011.01148.x 21631526

[32] Eildermann K Aeckerle N Debowski K: Developmental expression of the pluripotency factor sal-like protein 4 in the monkey, human and mouse testis: restriction to premeiotic germ cells. *Cells Tissues Organs.* 2012;196(3):206–220 10.1159/000335031 22572102

[33] Hobbs RM Fagoonee S Papa A: Functional antagonism between Sall4 and Plzf defines germline progenitors. *Cell Stem Cell.* 2012;10(3):284–298 10.1016/j.stem.2012.02.004 22385656 PMC3299297

[34] Nagamatsu G Kosaka T Saito S: Tracing the conversion process from primordial germ cells to pluripotent stem cells in mice. *Biol Reprod.* 2012;86(6):182 10.1095/biolreprod.111.096792 22423052

[35] Culty M: Gonocytes, the forgotten cells of the germ cell lineage. *Birth Defects Res C Embryo Today.* 2009;87(1):1–26 10.1002/bdrc.20142 19306346

[36] van de Geijn GJ Hersmus R Looijenga LH: Recent developments in testicular germ cell tumor research. *Birth Defects Res C Embryo Today.* 2009;87(1):96–113 10.1002/bdrc.20140 19306344

[37] Richardson BE Lehmann R: Mechanisms guiding primordial germ cell migration: strategies from different organisms. *Nat Rev Mol Cell Biol.* 2010;11(1):37–49 10.1038/nrm2815 20027186 PMC4521894

[38] Bendel-Stenzel M Anderson R Heasman J: The origin and migration of primordial germ cells in the mouse. *Semin Cell Dev Biol.* 1998;9(4):393–400 10.1006/scdb.1998.0204 9813186

[39] Farini D La Sala G Tedesco M: Chemoattractant action and molecular signaling pathways of Kit ligand on mouse primordial germ cells. *Dev Biol.* 2007;306(2):572–583 10.1016/j.ydbio.2007.03.031 17467686

[40] Gu Y Runyan C Shoemaker A: Steel factor controls primordial germ cell survival and motility from the time of their specification in the allantois, and provides a continuous niche throughout their migration. *Development.* 2009;136(8):1295–1303 10.1242/dev.030619 19279135

[41] Molyneaux KA Zinszner H Kunwar PS: The chemokine SDF1/CXCL12 and its receptor CXCR4 regulate mouse germ cell migration and survival. *Development.* 2003;130(18):4279–4286 10.1242/dev.00640 12900445

[42] Mansour AA Gafni O Weinberger L: The H3K27 demethylase Utx regulates somatic and germ cell epigenetic reprogramming. *Nature.* 2012;488(7411):409–413 10.1038/nature11272 22801502

[43] De Felici M: Regulation of primordial germ cell development in the mouse. *Int J Dev Biol.* 2000;44(6):575–580 11061420

[44] Sekido R: SRY: A transcriptional activator of mammalian testis determination. *Int J Biochem Cell Biol.* 2010;42(3):417–420 10.1016/j.biocel.2009.12.005 20005972

[45] Kashimada K Koopman P: Sry: the master switch in mammalian sex determination. *Development.* 2010;137(23):3921–3930 10.1242/dev.048983 21062860

[46] Barsoum I Yao HH: The road to maleness: from testis to Wolffian duct. *Trends Endocrinol Metab.* 2006;17(6):223–228 10.1016/j.tem.2006.06.009 16822678 PMC4073594

[47] Kanai Y Hiramatsu R Matoba S: From SRY to SOX9: mammalian testis differentiation. *J Biochem.* 2005;138(1):13–19 10.1093/jb/mvi098 16046443

[48] Kocer A Reichmann J Best D: Germ cell sex determination in mammals. *Mol Hum Reprod.* 2009;15(4):205–213 10.1093/molehr/gap008 19218284 PMC2657314

[49] Kierszenbaum AL Tres LL: Primordial germ cell-somatic cell partnership: a balancing cell signaling act. *Mol Reprod Dev.* 2001;60(3):277–280 10.1002/mrd.1088 11599037

[50] Bowles J Koopman P: Retinoic acid, meiosis and germ cell fate in mammals. *Development.* 2007;134(19):3401–3411 10.1242/dev.001107 17715177

[51] Saga Y: Sexual development of mouse germ cells: Nanos2 promotes the male germ cell fate by suppressing the female pathway. *Dev Growth Differ.* 2008;50(Suppl 1):S141–S147 10.1111/j.1440-169X.2008.01009.x 18430166

[52] Oatley JM Brinster RL: Spermatogonial stem cells. *Methods Enzymol.* 2006;419:259–282 10.1016/S0076-6879(06)19011-4 17141059

[53] Reuter VE: Origins and molecular biology of testicular germ cell tumors. *Mod Pathol.* 2005;18(Suppl 2):S51–S60 10.1038/modpathol.3800309 15761466

[54] Giwercman A Giwercman YL: Environmental factors and testicular function. *Best Pract Res Clin Endocrinol Metab.* 2011;25(2):391–402 10.1016/j.beem.2010.09.011 21397206

[55] Cools M Looijenga LH Wolffenbuttel KP: Disorders of sex development: update on the genetic background, terminology and risk for the development of germ cell tumors. *World J Pediatr.* 2009;5(2):93–102 10.1007/s12519-009-0020-7 19718530

[56] Krausz C Looijenga LH: Genetic aspects of testicular germ cell tumors. *Cell Cycle.* 2008;7(22):3519–3524 10.4161/cc.7.22.6980 19001860

[57] Rajpert-de Meyts E Hoei-Hansen CE: From gonocytes to testicular cancer: the role of impaired gonadal development. *Ann N Y Acad Sci.* 2007;1120:168–180 10.1196/annals.1411.013 18184914

[58] Gilbert D Rapley E Shipley J: Testicular germ cell tumours: predisposition genes and the male germ cell niche. *Nat Rev Cancer.* 2011;11(4):278–288 10.1038/nrc3021 21412254

[59] Hussain SA Ma YT Palmer DH: Biology of testicular germ cell tumors. *Expert Rev Anticancer Ther.* 2008;8(10):1659–1673 10.1586/14737140.8.10.1659 18925857

[60] Rapley EA Turnbull C Al Olama AA: A genome-wide association study of testicular germ cell tumor. *Nat Genet.* 2009;41(7):807–810 10.1038/ng.394 19483681 PMC2871592

[61] Jessberger R: New insights into germ cell tumor formation. *Horm Metab Res.* 2008;40(5):342–346 10.1055/s-2008-1073168 18491254

[62] Rapley EA Crockford GP Easton DF: Localisation of susceptibility genes for familial testicular germ cell tumour. *APMIS.* 2003;111(1):128–133 10.1034/j.1600-0463.2003.11101171.x 12752252

[63] Liu L Ishihara K Ichimura T: MCAF1/AM is involved in Sp1-mediated maintenance of cancer-associated telomerase activity. *J Biol Chem.* 2009;284(8):5165–5174 10.1074/jbc.M807098200 19106100

[64] Lessel D Gamulin M Kulis T: Replication of genetic susceptibility loci for testicular germ cell cancer in the Croatian population. *Carcinogenesis.* 2012;33(8):1548–1552 10.1093/carcin/bgs218 22745383

[65] Joffe M: What has happened to human fertility? *Hum Reprod.* 2010;25(2):295–307 10.1093/humrep/dep390 19933234

[66] Bray F Ferlay J Devesa SS: Interpreting the international trends in testicular seminoma and nonseminoma incidence. *Nat Clin Pract Urol.* 2006;3(10):532–543 10.1038/ncpuro0606 17031378

[67] Looijenga LH Oosterhuis JW: Pathogenesis of testicular germ cell tumours. *Rev Reprod.* 1999;4(2):90–100 10.1530/ror.0.0040090 10357096

[68] Okamoto K Kawakami T: Epigenetic profile of testicular germ cell tumours. *Int J Androl.* 2007;30(4):385–392 10.1111/j.1365-2605.2007.00754.x 17521367

[69] Looijenga LH: Human testicular (non)seminomatous germ cell tumours: the clinical implications of recent pathobiological insights. *J Pathol.* 2009;218(2):146–162 10.1002/path.2522 19253916

[70] Heaney JD Anderson EL Michelson MV: Germ cell pluripotency, premature differentiation and susceptibility to testicular teratomas in mice. *Development.* 2012;139(9):1577–1586 10.1242/dev.076851 22438569 PMC3317965

[71] Beumer TL Roepers-Gajadien HL Gademan IS: Involvement of the D-type cyclins in germ cell proliferation and differentiation in the mouse. *Biol Reprod.* 2000;63(6):1893–1898 10.1095/biolreprod63.6.1893 11090462

[72] Gilbert DC Chandler I McIntyre A: Clinical and biological significance of CXCL12 and CXCR4 expression in adult testes and germ cell tumours of adults and adolescents. *J Pathol.* 2009;217(1):94–102 10.1002/path.2436 18839394

[73] Gillis AJ Stoop HJ Hersmus R: High-throughput microRNAome analysis in human germ cell tumours. *J Pathol.* 2007;213(3):319–328 10.1002/path.2230 17893849

[74] Looijenga LH Gillis AJ Stoop H: Relevance of microRNAs in normal and malignant development, including human testicular germ cell tumours. *Int J Androl.* 2007;30(4):304–314 10.1111/j.1365-2605.2007.00765.x 17573854

[75] Voorhoeve PM le Sage C Schrier M: A genetic screen implicates miRNA-372 and miRNA-373 as oncogenes in testicular germ cell tumors. *Cell.* 2006;124(6):1169–1181 10.1016/j.cell.2006.02.037 16564011

[76] McIver SC Roman SD Nixon B: miRNA and mammalian male germ cells. *Hum Reprod Update.* 2012;18(1):44–59 10.1093/humupd/dmr041 21989172

[77] McIver SC Stanger SJ Santarelli DM: A unique combination of male germ cell miRNAs coordinates gonocyte differentiation. *PLoS One.* 2012;7(4):e35553 10.1371/journal.pone.0035553 22536405 PMC3334999

[78] Di Vizio D Cito L Boccia A: Loss of the tumor suppressor gene PTEN marks the transition from intratubular germ cell neoplasias (ITGCN) to invasive germ cell tumors. *Oncogene.* 2005;24(11):1882–1894 10.1038/sj.onc.1208368 15674339

[79] Gilbert DC McIntyre A Summersgill B: Minimum regions of genomic imbalance in stage I testicular embryonal carcinoma and association of 22q loss with relapse. *Gene Chromosomes Cancer.* 2011;50(3):186–195 10.1002/gcc.20843 21213372

[80] Freemantle SJ Vaseva AV Ewings KE: Repression of cyclin D1 as a target for germ cell tumors. *Int J Oncol.* 2007;30(2):333–340 17203214

[81] Orom UA Lund AH: Isolation of microRNA targets using biotinylated synthetic microRNAs. *Methods.* 2007;43(2):162–165 10.1016/j.ymeth.2007.04.007 17889804

[82] Basciani S De Luca G Dolci S: Platelet-derived growth factor receptor beta-subtype regulates proliferation and migration of gonocytes. *Endocrinology.* 2008;149(12):6226–6235 10.1210/en.2008-0349 18687785

[83] Takashima S Kanatsu-Shinohara M Tanaka T: Rac mediates mouse spermatogonial stem cell homing to germline niches by regulating transmigration through the blood-testis barrier. *Cell Stem Cell.* 2011;9(5):463–475 10.1016/j.stem.2011.08.011 22056142

[84] Thuillier R Mazer M Manku G: Interdependence of platelet-derived growth factor and estrogen-signaling pathways in inducing neonatal rat testicular gonocytes proliferation. *Biol Reprod.* 2010;82(5):825–836 10.1095/biolreprod.109.081729 20089883 PMC2857630

[85] Jiang W Xiang C Cazacu S: Insulin-like growth factor binding protein 7 mediates glioma cell growth and migration. *Neoplasia.* 2008;10(12):1335–1342 19048112 10.1593/neo.08694 PMC2586684

[86] Amemiya Y Yang W Benatar T: Insulin like growth factor binding protein-7 reduces growth of human breast cancer cells and xenografted tumors. *Breast Cancer Res Treat.* 2011;126(2):373–384 10.1007/s10549-010-0921-0 20464481

[87] Chen D Yoo BK Santhekadur PK: Insulin-like growth factor-binding protein-7 functions as a potential tumor suppressor in hepatocellular carcinoma. *Clin Cancer Res.* 2011;17(21):6693–6701 10.1158/1078-0432.CCR-10-2774 21908579 PMC3207018

[88] Medeiros LA Dennis LM Gill ME: mir-290-295 deficiency in mice results in partially penetrant embryonic lethality and germ cell defects. *Proc Natl Acad Sci U S A.* 2011;108(34):14163–14168 10.1073/pnas.1111241108 21844366 PMC3161528

[89] Zheng GX Ravi A Calabrese JM: A latent pro-survival function for the mir-290-295 cluster in mouse embryonic stem cells. *PLoS Genet.* 2011;7(5):e1002054 10.1371/journal.pgen.1002054 21573140 PMC3088722

[90] Zovoilis A Pantazi A Smorag L: Embryonic stem cell-related miRNAs are involved in differentiation of pluripotent cells originating from the germ line. *Mol Hum Reprod.* 2010;16(11):793–803 10.1093/molehr/gaq053 20566704

[91] Yang Y Wu J Guan H: MiR-136 promotes apoptosis of glioma cells by targeting AEG-1 and Bcl-2. *FEBS Lett.* 2012;586(20):3608–3612 10.1016/j.febslet.2012.08.003 22967897

[92] Shi Q Gibson GE: Up-regulation of the mitochondrial malate dehydrogenase by oxidative stress is mediated by miR-743a. *J Neurochem.* 2011;118(3):440–448 10.1111/j.1471-4159.2011.07333.x 21623795 PMC3135703

[93] Rajpert-De Meyts E: Developmental model for the pathogenesis of testicular carcinoma *in situ*: genetic and environmental aspects. *Hum Reprod Update.* 2006;12(3):303–323 10.1093/humupd/dmk006 16540528

