# Supplementary material for: The rise of testicular germ cell tumours: the search for causes, risk factors and novel therapeutic targets
Source: F1000Res. 2013 Feb 19;2:55. [Version 1] doi: 10.12688/f1000research.2-55.v1 (PMC3901536; doi:10.12688/f1000research.2-55.v1)
Supplement: Peer review history

# Peer review: Referee response for version 1

**Published:** 2013-05-28

**Authors:** John Schjenken

**Affiliations:** 1 Centre for Reproductive Health, University of Adelaide, Adelaide, SA, Australia

This article is a review discussing testicular germ cell tumours and how they may be triggered with an emphasis on miRNA expression. There are a few minor grammatical and spelling errors throughout this review but these do not impact on the quality of the manuscript. I would like to point out however that my area of expertise is primarily miRNA biology and I do not have a great background in TGCT.

I have read this submission. I believe that I have an appropriate level of expertise to confirm that it is of an acceptable scientific standard.

# Peer review: Referee response for version 1

**Published:** 2013-02-21

**Authors:** Anis Feki

**Affiliations:** 1 Department of Obstetrics and Gynaecology, Geneva University Hospitals, Geneva, Switzerland

I have read this submission. I believe that I have an appropriate level of expertise to confirm that it is of an acceptable scientific standard.
